# Role of Laparoscopy in the Surgical Management of Acute Small Bowel Obstruction: Fact or Fiction?

**DOI:** 10.7759/cureus.18828

**Published:** 2021-10-16

**Authors:** Rafique Umer Harvitkar, Peri Harish Kumar, Abhijit Joshi

**Affiliations:** 1 General Surgery, Dr Lakhumal Hiranand Hiranandani Hospital, Mumbai, IND; 2 Department of Surgery, Armed Forces Medical College, Pune, IND; 3 Gastrointestinal and Endolaparoscopic Surgery, Dr Lakhumal Hiranand Hiranandani Hospital, Mumbai, IND

**Keywords:** acute, laparoscopic, small bowel obstruction, adhesions, peritonitis, conversion, ischemic bowel

## Abstract

Background: Laparotomy (open surgery) is considered the standard approach for acute small bowel obstruction (ASBO). However, with the advent of minimally invasive surgery, the laparoscopic approach is gaining popularity. There is no consensus on the appropriate setting for laparoscopic therapy for small bowel obstruction (SBO).

Aim and objectives:* *The purpose of this study is to evaluate the outcomes of laparoscopic surgery for ASBO.

Patients and methods: We retrospectively evaluated the prospectively collected data of all the 38 patients who had undergone laparoscopy for ASBO, performed by a single surgeon at our institution, due to adhesions (30 patients), internal hernias (five patients), midgut malrotation (one patient), ileo-ileal intussusception (one patient), and superior mesenteric artery (SMA) syndrome (one patient) from 2012 to 2020. Data were extracted from the hospital electronic medical records (EMR) for the following parameters of each individual patient: age, sex, clinical presentation, preoperative investigation findings, final diagnosis, surgical details, operating time, time to postoperative oral feeds, length of hospital stay, complications, recurrences, and time taken to resume normal activity. A preoperative abdominal contrast-enhanced computed tomography (CECT) was performed in all the cases. Patients with peritonitis and septic shock were excluded from the study.

Results: The mean age of the 38 patients was 58 years (ranged between 33 and 83 years) with a standard deviation (SD) of 16.5. The mean age of the female patients in the study was 60.5 years with an SD of 16.6, while the mean age of the male patients was 54.9 years (SD = 16.2). The age difference between male and female patients in the study was not statistically significant (p = 0.36). The mean operating time was 74.4 minutes (range: 60-90 minutes, with an SD of 7.2). The mean time to oral liquid/soft diet was 2.5 days. The mean postoperative stay was 5.7 days. Three patients (8%) underwent conversion to open surgery, out of which two patients had multiple complex bowel-to-bowel and bowel-to-parietes adhesions, and in one patient, massive distension of small bowel caused technical difficulties.

Conclusion: Laparoscopic management of ASBO is feasible, effective, and safe. Optimum surgical techniques, the surgeon's experience with the procedure, and stringent patient selection criteria enable a high probability of success.

## Introduction

Acute small bowel obstruction (ASBO) is one of the most common causes of emergency hospital admissions for acute abdominal pain. Postoperative intra-abdominal adhesions occur in 55%-80% of the cases with small bowel obstruction (SBO) [1,2]. Common symptoms of SBO are abdominal colicky pain, abdominal distension, constipation, obstipation in some, and vomiting. The clinical signs of SBO include tympanic abdominal distension, tenderness, hyperperistalsis, and empty rectum.

The clinical picture could be a combination of any or all of the above signs and symptoms. The relevant investigations include a plain x-ray of the abdomen in standing/erect position, which shows the dilated gas-filled small bowel loops and may show multiple air-fluid levels in characteristic "stepladder pattern" in the central abdomen. A contrast-enhanced computed tomography (CECT) of the abdomen helps in locating the level of obstruction in addition to diagnosing specific causative pathologies such as luminal tumors, extrinsic compression by masses, etc. Initial management includes a trial of conservative management with intravenous antibiotics, intravenous fluid support, close monitoring of vital parameters, continuous nasogastric suction, and nil per oral state, pending the investigational workup. 

Around 45% require surgical treatment [3]. The morbidity and mortality, post-laparoscopic interventions for ASBO, are 15.5% and 1.5%, respectively [4]. Conventional laparotomy with adhesiolysis and, if required, resection of the small bowel has been the standard approach if conservative measures fail to relieve the obstruction. However, genuine concern with the open surgical procedure is the high potential of even more adhesions, after the intervention.

Retrospective cohort studies have suggested that with a minimum follow-up for 10 years, around 33% of patients with prior abdominal or pelvic surgery (laparotomy) are admitted at least once for ASBO [1]. Laparoscopic surgery (LS) has demonstrated benefits in offering lower morbidity, less postoperative pain, short hospital stays, less adhesion formation, and faster recuperation [1,5]. Bastug et al. reported the first case of laparoscopic adhesiolysis for ASBO in 1991 [6]. There are considerable controversies regarding using the laparoscopic approach in ASBO because of the difficulty of handling distended bowel, constrained working space, fear of iatrogenic perforation, and presumably higher cost of the procedure [7]. However, in the guidelines published by the World Society of Emergency Surgery on adhesive SBO group, the only contraindication for the laparoscopic approach was related to pneumoperitoneum (hemodynamic instability or cardiopulmonary impairment) [8,9]. The lack of consensus on indications and contraindications for the role of LS in ASBO in existing world literature has meant that it remains a second option to open surgery. The purpose of this study is to evaluate and analyze the outcomes of laparoscopic surgical therapy for selected cases of ASBO. It aims to hopefully reinforce faith and belief in LS as the preferred "first choice" therapeutic modality in selected cases of ASBO, among the surgical fraternity.

## Materials and methods

Patients and methods 

**Figure 1:**
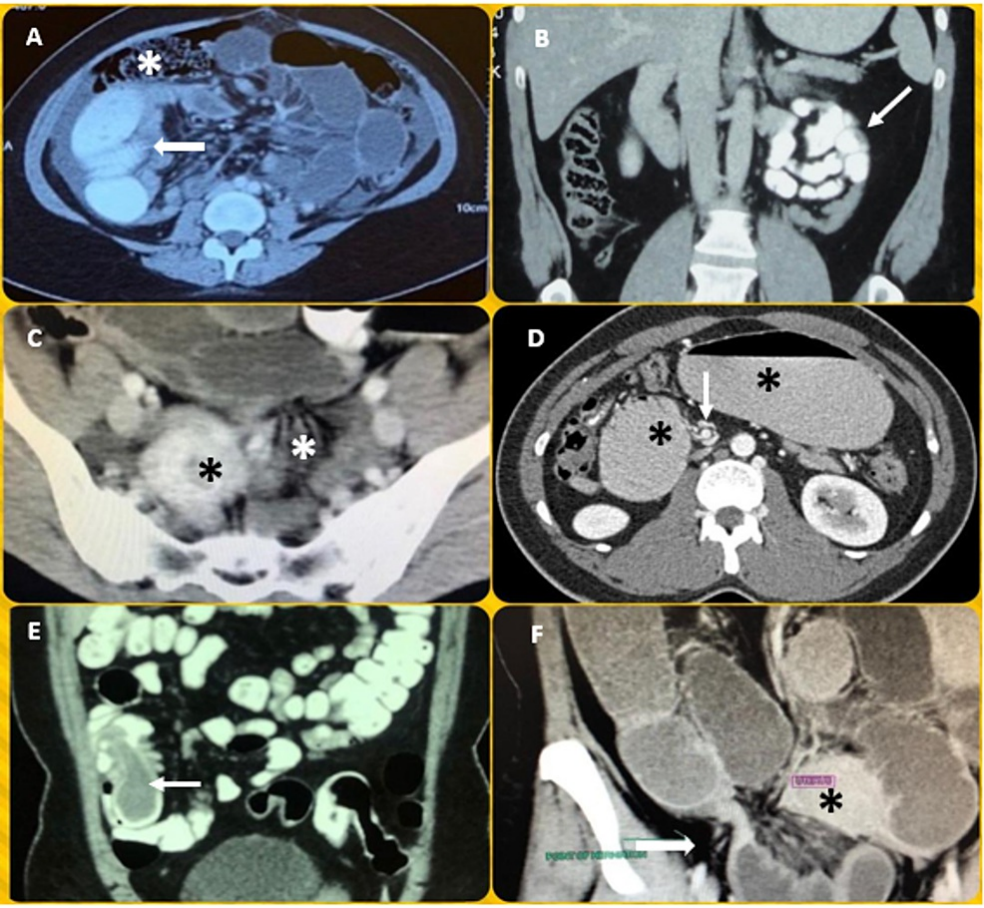
CECT of the abdomen appearances of some of the rare and exciting cases (A) Axial view of right PDH showing encapsulated contrast-filled dilated ileal loops (white arrow) posterior to ascending colon (white asterisk); (B) coronal view of left PDH showing a cluster of encapsulated contrast-filled ileal loops to the left of midline; (C) coronal view of left BLH showing herniated ileal loops through the broad ligament defect (white asterisk) pushing the uterus (black asterisk) a little to the right; (D) axial view of midgut malrotation showing the dilated stomach and duodenum (black asterisks) and the “whirlpool” sign of mesenteric vessels (white arrow); (E) coronal view of ileo-ileal intussusception revealing “sausage” sign (white arrow); and (F) coronal view of postoperative adhesion-related right-sided internal hernia showing point of transition (white arrow) on the right lateral side of the uterus (black asterisk). CECT, Contrast-enhanced computed tomography; PDH, paraduodenal hernia; BLH, broad ligament herniation.

**Figure 2:**
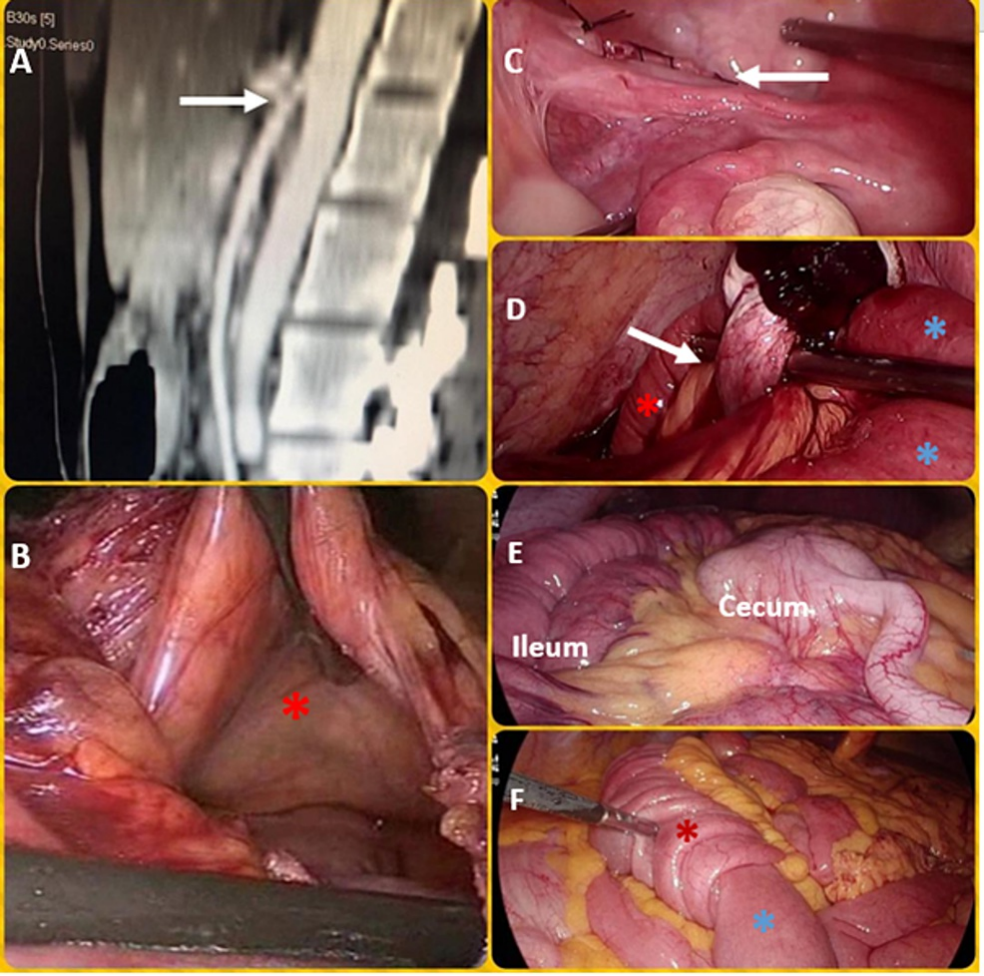
Operative pictures of the abdomen (A) CT angiography of the abdomen showing the severely reduced aortomesenteric angle (white arrow); (B-F) operative pictures. Panel (B) shows marsupialized fossa of Landzert of the left PDH (red asterisk). Panel (C) shows suture closed left BLH defect (white arrow). Panel (D) shows the second case of BLH-proximal dilated ileum (blue asterisks) and distal entrapped ileum (red asterisk) separated by left broad ligament defect (white arrow) with overlying left ovary and fallopian tube. Panel (E) shows midgut malrotation with the cecum and appendix medial to the ileum. Panel (F) shows ileo-ileal intussusception with intussusceptum (blue asterisk) telescoping into the intussuscipiens (red asterisk). PDH, Paraduodenal hernia; BLH, broad ligament herniation.

This study was conducted in adherence to the declaration of Helsinki. A retrospective review of patients diagnosed with SBO who underwent LS by a single surgeon at our institution from 2012 to 2020 was performed. The diagnosis of SBO was determined based on medical history, physical examination, and imaging modalities (x-ray of abdomen and CECT of abdomen and pelvis). Figures 1, 2 (Panel A) show the CECT of the abdomen appearances of some of the rare and exciting cases in this series.

The surgical approach was chosen as per the patient's general condition and diagnosis. Following the standard protocol, a nasogastric tube (NGT) was placed and kept open under regular suctioning when ASBO was suspected. The patient was kept nil per oral on intravenous fluid support and antibiotics. An oral and intravenous contrast-enhanced computed tomography (CECT) of the abdomen was then performed in all patients. Only those ASBO patients who satisfied the following criteria were subjected to LS: (1) patients with a good general condition, i.e., without hemodynamic instability or sepsis and (2) patients in whom there was no mass, tumor, or malignancy as the cause of SBO.

Our exclusion criteria for laparoscopic surgical interventions in ASBO patients were as follows: (1) patients with complicated intestinal obstruction, i.e., septic shock; (2) patients with suspected malignancies; (3) patients with suspected bowel strangulation, ischemia, necrosis, or perforation; (4) patients with severe cardiovascular compromise, significant respiratory or hemostatic pathologies; and (5) patients in whom the medical records were incomplete.

Data were collected from the hospital records for age, sex, ASA (American Society of Anesthesiology) grade, medical and surgical history, clinical presentation, radiological findings, surgical details (time, procedure, and result), the reason for the conversion - where applicable, time to initiation of diet (liquid or soft), perioperative complications, length of hospital stay, and mortality. As per departmental protocol, patients had been followed up in 10 days, three months, and one year after the surgery. Those patients who failed to follow up physically at three months and one year after the surgery had been interviewed telephonically at those respective times, and their responses were recorded electronically. This prospectively collected and recorded follow-up information was retrieved from the hospital EMR too. Thus, ours is a retrospective study of prospectively collected and recorded data. While writing this article, all the patients who were past their final postoperative outpatient department follow-up visits were also interviewed telephonically for any recurrence of symptoms.

Preoperative Preparation

Preoperative preparation was in the form of adequate intravenous fluid replenishment, maintaining normal electrolyte balance, judicial use of antibiotics, and appropriate antithrombotic prophylaxis. Nasogastric decompression was carried out in each patient. This facilitated decompression above the level of the obstruction, thereby minimizing the risk of aspiration during anesthesia and deflating the proximal distended bowel, which enabled relatively easier handling of the dilated bowel during surgery. Written, informed consent was obtained from every patient, prior to surgery.

Patient Position and the Surgical Technique

The patients were given a supine position with legs straight and split up. They were firmly strapped fixed to the table at the lower chest level to enable steep Trendelenburg, reverse Trendelenburg, and right and left lateral positions. The pressure points and contact areas were adequately padded. In patients who did not have scars of previous surgery on the abdomen, our preferred point of entry was the umbilical area (supra- or infraumbilical, depending on the adequacy of the umbilicus to pubis distance). If the bowel loops were massively distended and the abdomen was too tense, then we preferred to do a direct 10-mm blunt trocar insertion by the open technique in the umbilical area (either infra- or supraumbilical). In other situations, where the NGT had aspirated copious amounts and the abdomen was relatively soft, we preferred to institute pneumoperitoneum through the chosen site by the conventional Veress needle technique. In patients with scars of previous abdominal surgery, we preferred to institute pneumoperitoneum through the Veress needle at Palmer's point (a relatively safe point for entry, on the left midclavicular line two finger breadths below the costal margin). Then, a 5-mm trocar was inserted at the same point, and a peripheral bird's eye view of the abdomen was obtained through a 5-mm telescope that was inserted through this trocar. Central trocars were then inserted, carefully dodging any adhesions (if present), under the vision provided by this 5-mm telescope. Dense adhesions, if present, were first lysed through additional peripheral trocars inserted in "safe areas" before insertion of the central trocars.

Once the central 10-mm trocar was inserted, we switched over to a 10-mm telescope and inserted the suprapubic 10-mm trocar, which then became our primary optic trocar. An additional 5-mm trocar was inserted into the right iliac fossa (RIF). Once the telescope was shifted to the suprapubic trocar, the RIF and umbilical trocars become the surgeon's left and right-hand working ports, respectively. A systematic "bowel walk" was initiated, starting from the ileocecal junction to the duodenojejunal flexure. We believe that the suprapubic optic trocar provides an optimum view of the central abdomen, which is essentially the theater of SBO, thus enabling accurate identification and localization of the pathology and eventual therapy. While dealing with concurrent complicated adhesions and significant small bowel distension, we believe that one should not hesitate to insert one or two extra trocars at optimum places in order to insert extra instruments like the fan retractor for better atraumatic retraction of dilated bowel and safer surgery. In the SMA syndrome case, the suprapubic trocar was not required, and the umbilical trocar remains the optic trocar throughout the surgery. In the adhesive SBO cases, the midgut malrotation case, and the SMA syndrome case, the operating surgeon stood between the patient's legs with the monitor above the patient's left shoulder. In the PDH cases, the monitor was placed by the ipsilateral side of the patient, facing the surgeon standing between the patient's split legs. In the BLH cases and the obstructed internal hernia due to the post-LSCS (lower uterine segment cesarean section) adhesions case, the surgeon stood on the contralateral side with the monitor near the patient's ipsilateral foot end. The harmonic scalpel was our preferred energy source for these surgeries, and we found it invaluable, especially while working in restricted spaces. Any free fluid was aspirated and sent for routine microscopy, gram stain, amylase level, and SOS cytology. 

**Figure 3:**
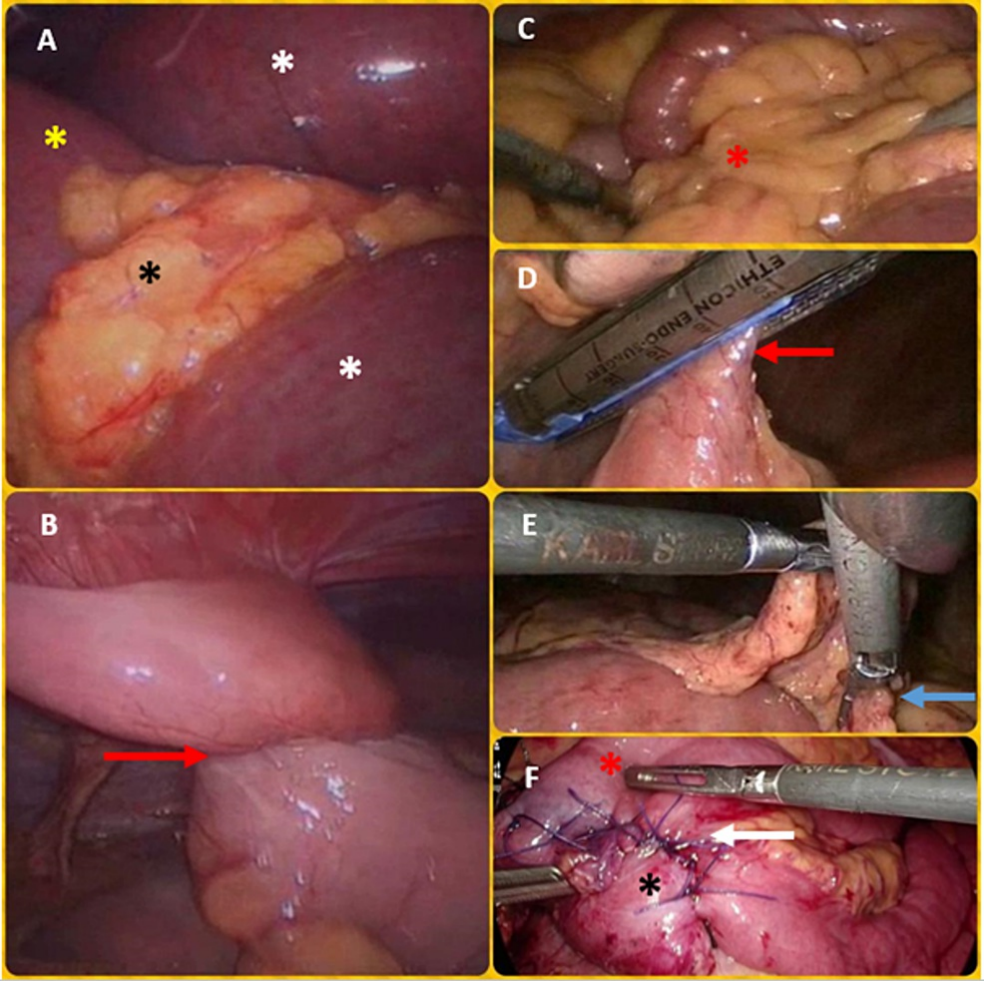
Operative pictures of the abdomen showing the procedures Panel (A) shows the obstructing omental band around the ileum (black asterisk) with dilated proximal ileum (white asterisk) and normal caliber distal ileum (yellow asterisk). Panel (B) shows an acute twist (red arrow) in the ileum adherent to the parietes at the site of previous surgery. Panel (C) shows the obstructing omental band (red asterisk) caused by greater omentum adherent to inflamed Meckel’s diverticulum. Panel (D) shows the excision of Meckel’s diverticulum using the stapler (red arrow). Panel (E) shows the obstructing omental band being lysed by a harmonic scalpel (blue arrow). Panel (F) shows the SMA syndrome case (white arrow points to D-J staple-suture line, red asterisk shows jejunum, and black asterisk shows D3). SMA, Superior mesenteric artery.

**Figure 4:**
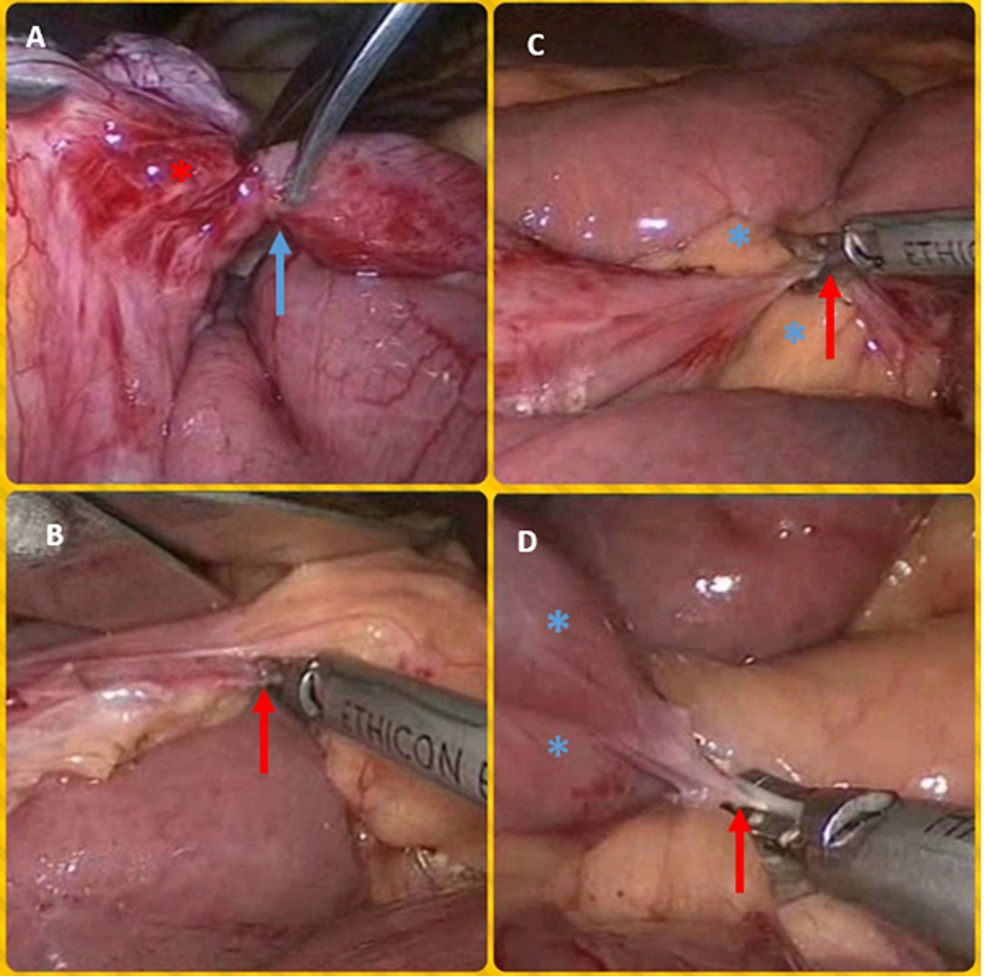
Pictures of operative procedures of the abdomen Panel (A) shows obstructing thick fibrotic band (red asterisk) being divided (blue arrow). Panel (B) shows the division of obstructing fibro-omental band using a harmonic scalpel (red arrow). Panel (C) shows the division of the fibrotic loop-to-loop band (red arrow) overlying mesentery of the ileum (blue asterisks). Panel (D) shows the division of a fibrotic loop-to-mesentery band (red arrow) causing an acute kink in the ileal loop (blue asterisks) to which it was attached (B-D are pictures of the same case of complicated multiple bands/adhesions).

A pair of "cold" scissors were used to divide the adhesions or bands. We usually avoided the use of energy sources nearby to the bowel. Extreme care was taken to minimize direct handling of distended bowel. Where needed, atraumatic "soft" bowel graspers were used. In case of iatrogenic bowel injury or evidence of ischemic/necrotic bowel, conversion to laparotomy was carried out. The bands were lysed in the adhesive SBO and midgut malrotation cases (Figure 2 [Panel E], Figure 3 [Panels A-E], Figure 4). In the right and left PDH cases, the abnormal recesses into which the small bowel was trapped (the fossae of Waldeyer and Landzert, respectively) were laid open (Figure 2, Panel B). In one of the BLH cases, the defect in the broad ligament was marsupialized due to the inability to reduce the hugely dilated and trapped ileum by gentle taxis (Figure 2, Panel D). In the second BLH case and the postoperative adhesions related to the internal hernia case, the defects were suture closed after completely reducing the entrapped small bowel (Figure 2, Panel C). In the latter, due to the inability to reduce entrapped small bowel by taxis, the defect was first carefully widened using the harmonic scalpel before reducing bowel and eventual suture closure. In the SMA syndrome case, a stapled cum sutured side-to-side duodeno-jejunostomy was performed (Figure 3, Panel F). In the ileo-ileal intussusceptions case, laparoscopic segmental resection of the involved segment was performed (Figure 2, Panel F). We left one liter of low molecular weight dextran (Lomodex) solution in the peritoneal cavity as an anti-adhesion barrier at the end of all these surgeries. 

## Results

Results of analysis of data retrieved of a total of 38 patients who had undergone LS for ASBO due to adhesions, internal hernias, intussusceptions, midgut malrotation, and SMA syndrome, from 2012 to 2020, are given in Table 1. They included 21 men (57%, with a mean age of 60.5 years, SD of 16.6) and 17 women (43%, with a mean age of 54.9 years, SD of 16.2) with a mean age of 58 years (range: 33-83, SD: 16.5). Among them, 30 patients (79%) had one or multiple adhesions or bands as the cause of obstruction. Of the remaining eight (21%), five patients (13%) had an internal hernia, while the remaining three patients (8%) had intussusception, midgut malrotation, and SMA syndrome, respectively. According to ASA classification, eight patients were graded I, 20 were II, nine were III, and one was IV. The patient demographics, complications after surgery, and length of stay have been summarized (Table 1). Eighteen (47%) patients had no history of previous surgery. Out of the remaining 20 (53%) patients who had a history of previous abdominal and/or pelvic surgery, 14 (70%) patients had been operated upon once, five (25%) patients had been operated upon twice, and one (5%) patient had been operated upon four times. Out of five patients (13%) operated for internal hernia, two had obstructed paraduodenal hernias (one each side), two had obstructed left broad ligament hernia, and one had an obstructed internal hernia caused by entrapment of ileum in an abnormal recess (most likely caused by post lower segment cesarean section adhesions). One patient had ileo-ileal intussusception, and two presented with acute duodenal obstruction caused by midgut malrotation and SMA syndrome.

**Table 1:** Summary of the patient demographics, complications after surgery, and length of stay

Parameters		N (%)
Total no. of patients		38
Mean age (range) in years		60 (33-83)
Sex	Male	21 (57%)
	Female	17 (43%)
Previous history of abdominal surgery	Yes	20 (54%)
	No	18 (46%)
Intraoperative course	Completed laparoscopically	35 (92%)
	Converted to open	03 (8%)
Intestinal resection	Yes	1 (2.7%)
Intra- and postoperative complications	Aspiration pneumonia	0
	Bowel perforation	0
	Surgical site adverse events	3 (8%)
	Recurrence of obstruction	1 (2.7%)
Hospitalization (days)	Completed laparoscopically	5-7 days
	Converted to open	11 days

The different causes of ASBO among all the patients of our series and the respective patient numbers and specific procedures performed have been summarized in Table 2. Three patients (8%) had a conversion to open surgery due to technical difficulties. All of these three patients had a history of the previous laparotomy. None of the group of patients with no previous history of open abdominal surgery underwent conversion to open surgery. In spite of this, due to low numbers of patients who converted to open surgery (n = 3), the p-value (> 0.05), signifying co-relation between the history of previous open abdominal surgery and conversion to open surgery, was not statistically significant. Among the patients who underwent conversion to laparotomy, two patients had mid-midline scars of previous emergency laparotomy for duodenal ulcer perforation and typhoid ileal perforation, respectively. The third patient had a left subcostal incision scar of a previous emergency splenectomy performed for accidental blunt abdominal trauma. Two out of the three patients who converted to open surgery had multiple and complex bowel-to-bowel and bowel-to-parietes adhesions, one of whom underwent a segmental resection and the other underwent adhesiolysis. The third patient had massive small bowel distension, which precluded the continuation of laparoscopy. He turned out to have a thick obstructing band over his terminal ileum, which was lysed at laparotomy. One patient had recurrent SBO after laparoscopic adhesiolysis, one month after his surgery. He was re-admitted and underwent a laparotomy with segmental ileal resection during the same.

**Table 2:** The different causes of ASBO among all the patients and the respective number of patients as well as the procedures performed ASBO, Acute small bowel obstruction.

Etiology of Obstruction	N (%)	Surgery Performed
Primary solitary adhesion/band	20 (52.6%)	Adhesiolysis
Postoperative solitary adhesion	6 (16%)	Adhesiolysis
Primary complex adhesion	3 (8%)	Adhesiolysis
Postoperative complex adhesion	1 (2.6%)	Adhesiolysis
Congenital internal hernia: right paraduodenal hernia	1 (2.6%)	Marsupialization
Congenital internal hernia: left paraduodenal hernia	1 (2.6%)	Marsupialization
Congenital midgut malrotation	1 (2.6%)	Ladd’s procedure
Superior mesenteric artery syndrome	1 (2.6%)	Duodeno-jejunostomy
Postoperative internal hernia: left broad ligament hernia	2 (5.2%)	One marsupialization and one suture closure of the defect
Postoperative internal hernia: postoperative	1 (2.6%)	Suture closure of the defect
Other causes: intussusception	1 (2.6%)	Segmental resection with ileo-ileal anastomosis

The mean operating time in this series was 74.4 minutes (range: 60-90 minutes, SD: 7.2). The mean operating time in the group with no previous history of open abdominal surgery (n = 18) was 73.9 minutes (SD: 8.2). The mean operating time in the group of patients with a history of open abdominal surgery (n = 20) was 74.8 minutes (SD: 6.4). With a p-value of 0.074, this correlation between operating time and history of previous open abdominal surgery was not statistically significant. The mean time to initial oral liquid diet intake was 2.5 days. The mean postoperative stay was six days. The time taken to resume routine activity was seven to 10 days. In the immediate postoperative period, mild complications were seen in five patients (14%). Three patients (8%) had trocar site wound erythema (grade IIa/IIb, respectively, as per the Southampton wound grading system), while two patients (5%) had mild trocar site bruising (grade I). In all the five patients, the said wound-related events were self-resolved on day 10 of follow-up. The histopathology report for resected bowel in the patient with intussusception turned out to reveal a benign inflammatory fibroid polyp at the lead point of the intussusceptum.

## Discussion

Acute intestinal obstruction is defined as sudden interruption of the forward flow of bowel contents. Intestinal obstruction comprises 15% of all the causes of acute abdominal pain for which patients present to the emergency department [10]. The causes of mechanical intestinal obstruction are categorized into extrinsic, intraluminal, and intramural. Extrinsic causes are the most common etiologies for ASBO. They include intraperitoneal adhesions (55%-75%), cancer (18%), hernia - external and internal (15%-20%), intussusception, and others (5%) [11]. Intraluminal causes include foreign bodies, biliary stones, bezoars (1%-4%), and parasites. Intestinal inflammation, fibrosis, stenosis, or ischemia may lead to impaired patency of the gastrointestinal tract and comprise the intramural causes, as also large polyps and/or cancers. Only 5% of ASBO patients have intramural causes [11-13]. Conventional laparotomy with adhesiolysis or bowel resection is the standard procedure for SBO if conservative methods fail or suspected complications such as ischemia, necrosis, or gangrene. However, a potential problem with open surgery is that it causes increased adhesions. Recently, minimally invasive surgery has been considered an alternative treatment for ASBO since it is associated with less postoperative adhesions [13]. The reason for not adopting the laparoscopic approach primarily in the past was a risk of iatrogenic intraoperative bowel injury, difficulty in handling dilated bowel loops, and limited working space due to dilated bowel loops. Hence, ASBO was considered a contraindication for laparoscopy. However, with the advancement of time, better instrumentation, better optics, and more evolved techniques; it is now only a relative contraindication [2,14].

Bastug et al. first reported successful laparoscopic adhesiolysis in 1991 for ASBO [6]. Since then, with technological progress and increased experience in minimally invasive procedures, many studies have shown the laparoscopic approach's feasibility, safety, and possible preferential role for treating ASBO [2,12-15]. There are no specific consensus guidelines on the indications for LS and ASBO. The current role of laparoscopy in ASBO has three broad dimensions. Firstly, laparoscopy is a definitive diagnostic tool (where imaging has not diagnosed a cause). Secondly, with laparoscopy, it is possible to locate the cause of the disease accurately and, if required, perform a mini-laparotomy in the afflicted abdominal quadrant under the vision and complete the surgery. Lastly, laparoscopy, with all its well-known advantages, may be used as the definitive therapeutic modality [14-16]. Conventional open surgery for ASBO is associated with high morbidity and prolonged hospitalization.

Wullstein et al., in their comparative study between laparoscopic and open approaches for SBO, obtained noteworthy differences in favor of laparoscopy. They found a lower rate of postoperative complications (20% vs. 35%), faster resumption of peristalsis (3.5 vs. 4.5 days), and shorter hospital stay (11.3 vs. 18 days) [13]. In a similar study, Khaikin et al. discovered the same results [17]. In their systemic review comparing laparoscopic vs. open adhesiolysis for SBO, Sajid et al. concluded that laparoscopic procedure reduces the risk of morbidity, mortality, and surgical site infection while shortening hospital stay [18]. The results of our study are generally consistent with previous reports in favor of LS. Most conversions to open surgery are due to technical difficulties and the inability to identify the cause of the obstruction. We believe that patients with single adhesion/band and internal hernias without the need for bowel resection are ideal candidates for laparoscopy.

The results of our study show that the laparoscopic approach in the management of ASBO is associated with better postoperative outcomes (14% minor complication rate), an earlier onset of oral intake (mean: 3 days, range: 4-6 days), and a shorter length of hospital stay (mean: 5 days, range: 7-11 days).

Although our results are favorable toward laparoscopy, we do not think that they are comparable to a group of patients for whom we would prefer open surgery because that group would present with more severe symptoms (septic shock, hemodynamic instability, cardiopulmonary impairment, bowel ischemia, or perforation). Its small size and retrospective nature also limit our study. Despite these limitations, the results obtained are strong enough to substantiate and underscore the benefits of the laparoscopic approach for carefully selected patients of ASBO.

## Conclusions

ASBO, once a contraindication for laparoscopy, is the latest frontier to come under its ambit. Development of optic technology, surgical instrumentation, and experience have collectively enabled this. As seen through this series, various causes of ASBO, such as solitary obstructing bands and, in some cases, multiple adhesive obstructing bands, internal hernias, intussusception, midgut malrotation, and SMA syndrome can be successfully treated totally laparoscopically. Past history of open abdominal surgery is not a contraindication for laparoscopy. Operating times and chances of conversion to open surgery increase slightly with a history of previous open abdominal surgery, though not to a statistically significant extent. In cases where optimum visualization and "bowel walk" are a challenge, it is better to insert an extra trocar or two for safe atraumatic retraction of distended bowel, thereby facilitating the surgery. Adhering to the time-honored principle of "*primum non nocere*," there should be very limited and careful usage of energy sources in the restricted working space of an abdomen with ASBO. Lastly, very delicate handling of distended, inflamed, and friable bowel with "soft," non-traumatic graspers is key to avoid iatrogenic bowel injury.
